# Racial and Ethnic Disparities in Adult Obesity in the United States: CDC’s Tracking to Inform State and Local Action

**DOI:** 10.5888/pcd16.180579

**Published:** 2019-04-11

**Authors:** Ruth Petersen, Liping Pan, Heidi M. Blanck

**Affiliations:** 1 National Center for Chronic Disease Prevention and Health Promotion, Centers for Disease Control and Prevention, Atlanta, Georgia

The Centers for Disease Control and Prevention (CDC) plays a key role in tracking data on the burden of obesity and its related racial and ethnic disparities to provide information that can highlight areas where state and local actions are most needed. Until further innovations allow for measured data on height and weight to be available for all states, self-reported data are the best source for understanding where the burden of obesity is highest among different populations. This understanding is critical given that the prevalence of obesity is increasing among adults in the United States (1). As such, obesity continues to put a strain on overall health status, health care costs, productivity, and the capacity for deployment and readiness of military personnel. Adults with obesity often have multiple-organ system complications from the condition and, as a result, are more at risk for heart disease, stroke, type 2 diabetes, and multiple types of cancers (2). The estimated annual medical cost of obesity in the United States was $147 billion in 2008 (3). Compared with spending for someone of normal weight, medical spending for a person with obesity was $1,429 higher (42% higher) per year (3). Adult obesity decreases productivity, and the cost of lost productivity is between $3.4 and $6.4 billion per year (4). Adult obesity also increases the risk of workplace injuries (2). Obesity among young adults limits the eligibility for many to serve in our military, given the weight standards for recruitment that nearly 1 in 4 young adults are not able to meet (5).

Among many other factors, the risk of adult obesity is greater among adults who had obesity as children, and racial and ethnic disparities exist by the age of 2 (6). If nothing else is done in the United States beyond what is being done now, simulated growth trajectories that model today’s children show that over half (59% of today’s toddlers and 57% of children aged 2 to 19) will have obesity at age 35 (7). Early feeding patterns, including how babies are fed and how caregivers use food in response to an infant’s mood, affect acute growth, future eating patterns, and the risk of obesity (8). Similarly, family and caregiver modeling of healthy behaviors, food offerings, and active playtime, as well as characteristics of neighborhoods such as walkability and traffic volume, may affect children’s nutrition and physical activity habits (9,10).

As sectors come together to reduce the obesity epidemic, we are aware how challenging success will be due to factors such as 1) the contributing risk factors of genetic and biological attributes; 2) individual behaviors (parenting styles, dietary patterns, physical activity levels, medication use, sleep, stress management); and 3) community and societal factors that influence individual, family, and collective access to healthy, affordable foods and beverages; access to safe and convenient places for physical activity; and exposure to the marketing of unhealthy products (2).

By using self-reported data of height and weight from the Behavioral Risk Factor Surveillance System, CDC’s Division of Nutrition, Physical Activity, and Obesity (DNPAO) has published state-specific obesity maps since 1999. Obesity is defined as a body mass index (a person’s weight in kilograms divided by the square of height in meters) of 30.0 or higher. These maps have shown the growing epidemic that has affected our nation from coast to coast. Although the data collection methods changed in 2011, which somewhat limits our ability to assess trends, the 2017 data continue to show that obesity prevalence among adults remains high across the country (Figure 1). The state-specific prevalence ranges from a low of 22.6% in Colorado to a high of 38.1% in West Virginia (11).

**Figure 1:**
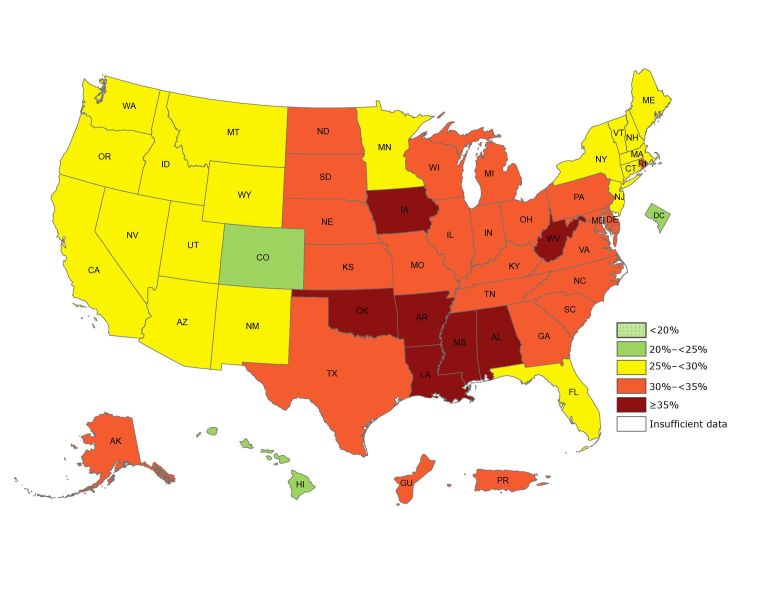
Prevalence of self-reported obesity among US adults, by state and territory, Behavioral Risk Factor Surveillance System (BRFSS), 2017. Obesity was defined as a body mass index of 30 or higher based on self-reported weight in kilograms divided by the square of the height in meters. Prevalence estimates reflect changes in BRFSS methods that started in 2011. These estimates should not be compared to prevalence estimates before 2011. No area had a prevalence of <20%, and all had sufficient data to determine prevalence.

**Table:** 

State or Territory	Percentage
Alabama	36.3
Alaska	34.2
Arizona	29.5
Arkansas	35.0
California	25.1
Colorado	22.6
Connecticut	26.9
Delaware	31.8
District of Columbia	22.9
Florida	28.4
Georgia	31.6
Guam	34.3
Hawaii	23.8
Idaho	29.3
Illinois	31.1
Indiana	33.6
Iowa	36.4
Kansas	32.4
Kentucky	34.3
Louisiana	36.2
Maine	29.1
Maryland	31.3
Massachusetts	25.9
Michigan	32.3
Minnesota	28.4
Mississippi	37.3
Missouri	32.5
Montana	25.3
Nebraska	32.8
Nevada	26.7
New Hampshire	28.1
New Jersey	27.3
New Mexico	28.4
New York	25.7
North Carolina	32.1
North Dakota	33.2
Ohio	33.8
Oklahoma	36.5
Oregon	29.4
Pennsylvania	31.6
Puerto Rico	32.9
Rhode Island	30.0
South Carolina	34.1
South Dakota	31.9
Tennessee	32.8
Texas	33.0
Utah	25.3
Vermont	27.6
Virginia	30.1
Washington	27.7
West Virginia	38.1
Wisconsin	32.0
Wyoming	28.8

For the past 4 years, CDC has published more detailed state and territorial maps that combine 3 years of data to create stable estimates of self-reported adult obesity by race/ethnicity. These maps help demonstrate the geographic and racial/ethnic disparities in obesity burden. Although the previously released overall state-specific maps demonstrate where obesity may be influencing health, health care costs, well-being, and productivity across states and regions, the racial and ethnic maps for 2015 through 2017 illustrate that the negative effects are disproportionately burdensome for particular populations. Combined data for 2015 through 2017 allowed for assessment by major racial/ethnic categories and found that non-Hispanic black adults had the highest prevalence of obesity (38.4%) overall, followed by Hispanic adults (32.6%) and non-Hispanic white adults (28.6%). To identify areas of highest burden, we used a cut point of 35%. We chose this cut point because it was a somewhat natural breaking point in the data and roughly reflected areas with the highest burden. By using this cut point, we found that overall, 31 states and the District of Columbia had an obesity prevalence of 35% or higher among non-Hispanic black adults; 8 states had an obesity prevalence of 35% or higher among Hispanic adults; and only 1 state had an obesity prevalence of 35% or higher among non-Hispanic white adults (Figure 2).

**Figure 2:**
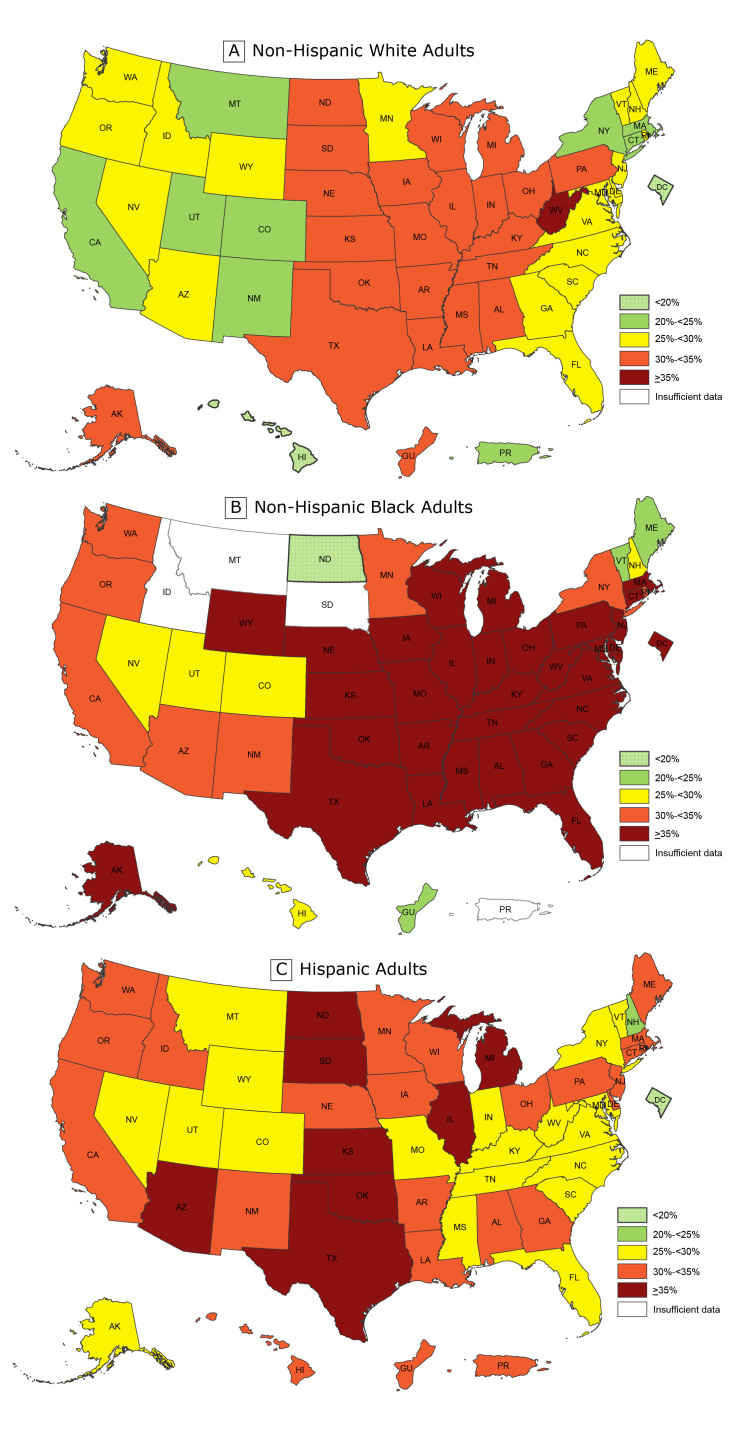
Prevalence of self-reported obesity among non-Hispanic white, non-Hispanic black, and Hispanic adults, by state and territory, Behavioral Risk Factor Surveillance System, 2015–2017. Obesity was defined as a body mass index of 30 or higher based on self-reported weight in kilograms divided by the square of the height in meters. Prevalence estimates reflect changes in BRFSS methods that started in 2011. These estimates should not be compared to prevalence estimates before 2011. Areas are indicated as having insufficient data if they had a sample size of less than 50 or a relative standard error (dividing the standard error by the prevalence) of 30% or more.

**Table:** 

State or Territory	Non-Hispanic White, %	Non-Hispanic Black, %	Hispanic, %
Alabama	33.1	45.0	31.9
Alaska	30.0	44.7	28.8
Arizona	26.1	32.4	35.5
Arkansas	34.0	44.2	30.1
California	23.1	31.4	32.1
Colorado	20.3	28.9	27.4
Connecticut	24.4	37.1	31.8
Delaware	29.7	37.4	31.9
District of Columbia	10.3	36.3	19.7
Florida	26.2	35.4	28.1
Georgia	29.5	37.1	30.1
Guam	30.2	22.8	33.0
Hawaii	17.5	29.8	31.9
Idaho	27.8	Insufficient data	33.7
Illinois	30.3	39.5	35.9
Indiana	32.1	42.2	28.2
Iowa	33.6	36.3	33.4
Kansas	32.0	41.2	36.8
Kentucky	34.4	40.2	28.5
Louisiana	33.4	42.6	32.3
Maine	29.8	24.8	32.2
Maryland	28.1	39.1	27.6
Massachusetts	24.0	35.1	31.0
Michigan	30.9	39.9	38.6
Minnesota	27.5	30.4	33.3
Mississippi	32.1	45.4	29.2
Missouri	31.6	39.1	29.9
Montana	24.0	Insufficient data	26.0
Nebraska	31.7	39.9	32.8
Nevada	25.7	29.2	29.2
New Hampshire	27.4	25.9	24.1
New Jersey	25.7	36.4	31.9
New Mexico	24.3	31.2	31.2
New York	24.7	33.4	28.7
North Carolina	29.3	41.1	28.3
North Dakota	31.9	19.6	36.5
Ohio	31.2	37.5	31.9
Oklahoma	33.4	37.6	36.8
Oregon	29.3	30.8	34.9
Pennsylvania	30.1	36.8	34.7
Puerto Rico	24.1	Insufficient data	31.0
Rhode Island	26.9	31.8	33.1
South Carolina	29.6	42.0	27.8
South Dakota	30.0	Insufficient data	35.0
Tennessee	31.8	46.4	29.6
Texas	30.1	39.8	37.9
Utah	24.7	26.3	27.9
Vermont	26.7	22.8	26.4
Virginia	27.9	41.0	29.9
Washington	28.3	33.7	33.9
West Virginia	37.0	43.6	29.0
Wisconsin	31.0	38.1	31.5
Wyoming	27.9	44.8	28.8

## What Causes These Disparities?

Although the exact causes of these differences in obesity are not all known, they likely in part reflect differences in social and economic advantage related to race or ethnicity (12). This concept aligns with other, more general statements about health disparities explaining that disparities are “closely linked with social, economic, and/or environmental disadvantage” and show the effect where groups of people “have systematically experienced greater social and/or economic obstacles to health . . . based on their racial or ethnic group” (13). Underlying risks that may help explain disparities in obesity prevalence among non-Hispanic black and the Hispanic populations could include lower high school graduation rates, higher rates of unemployment, higher levels of food insecurity, greater access to poor quality foods, less access to convenient places for physical activity, targeted marketing of unhealthy foods, and poor access to health care or referrals to convenient community organizations that aid family-management or self-management resources (14–17).

## What Is DNPAO Doing to Address These Disparities?

From a large number of high-quality applicants, in 2018 DNPAO competitively funded 16 state health departments (or a similar entity), 15 land grant colleges and universities, and 31 community-focused grantees to work over the course of 5 years with multiple sectors and coalitions to prioritize and implement best practices to increase healthy eating and active living to prevent obesity and other chronic diseases. With technical assistance from DNPAO public health specialists and subject matter experts, grantees use a menu of evidence-based strategies and performance metrics to develop their implementation plan, work plan, and evaluation process. To obtain the largest public health impact from limited resources, grantees are asked to focus their work on populations that have the greatest disparities and needs. Strategies for DNPAO grantees include establishing healthy nutrition standards in settings such as workplaces, hospitals, early care and education (ECE), after-school and recreational programs, and faith-based organizations; working with food vendors, distributors, and producers to increase procurement and sales of healthier foods; improving programs and systems at the state and local level to increase access to healthier food; and implementing community planning and transportation plans that support safe and accessible physical activity by connecting sidewalks, paths, bike routes, public transit with homes, ECE, schools, parks and recreation centers, and other everyday destinations.

As an example of reaching vulnerable individuals, state health department grantees may focus obesity prevention efforts at a state level by targeting early obesity risk through system changes in the ECE setting through state licensing, state subsidy, or state quality rating systems. States may pair these efforts with promoting the use of food reimbursement programs for meals that meet minimum nutritional standards among centers serving low-income children. In addition, state health departments may work to set a standard for implementation of food service guidelines so other government entities, work sites, park and recreation centers, and hospitals can follow that example and obtain the needed technical assistance for spreading implementation. State health department grantees may also work across sectors (such as the transportation and community planners) to improve environmental supports for physical activity through the implementation of master plans and land-use interventions. These efforts to increase access to safe and convenient places for physical activity are generally targeted to geographical areas with the highest burden of obesity and chronic disease. Such efforts can include connecting neighborhoods with sidewalks, paths, bike routes, and public transit that lead to local schools, parks and recreation centers, and local businesses.

DNPAO manages 2 additional public health practice programs that have had success in reducing the risk factors for obesity in populations with the greatest disparities. These programs include the Racial and Ethnic Approaches to Community Health (REACH) program and the High Obesity Program (HOP). The REACH program focuses on improving health for racial and ethnic groups with the highest disease burden. Obesity reduction among the black population is often a key goal for REACH recipients. For example, from 2008 through 2012, 14 REACH grantees implemented strategies to address disparities in obesity among black populations. These strategies included expanding healthy food choices in grocery stores, creating neighborhood farmers markets, implementing Complete Street policies, and improving walkability and safety of neighborhood streets. The prevalence of obesity decreased about 1 percentage point in these REACH communities, but not in the comparison populations during the same time (18).

Land grant universities in states where counties have more than a 40% prevalence of adult obesity are eligible to apply for HOP. These grantees work in predominantly rural areas where residents may have less access to healthy foods and fewer opportunities to be physically active, which may increase their risk of obesity (19–21). HOP grantees use the same menu of DNPAO evidence-based strategies to improve nutrition and physical activity to reduce obesity and other chronic diseases; however, they might tailor their implementation plan given the rural nature of their target population with the highest risk of obesity. Examples include work at the Texas AgriLife Extension (Texas A&M University), which established a farmers market at a local community center to help increase access to fresh produce. Since the creation of this market, more than 800 community members purchased over 12,000 pounds of fresh fruits and vegetables. Another example is the work of the extension staff in Ouachita County (University of Arkansas) at a low-income housing complex to improve access to physical activity for residents with limited mobility. They identified a walking path and developed signs to indicate how many laps equaled a half-mile. Eighty-four percent of residents now walk regularly and use the path at least 1 or 2 times a week (22).

## What’s Next?

Implementing approaches that take into account racial and ethnic disparities is critical to addressing the high burden of obesity and its many negative consequences. Although a population-based approach is needed to increase availability and access to healthy foods and beverages and safe and convenient places for physical activity for all Americans, targeted approaches are needed to address the risks that drive the disparities. Such an approach will mean taking into account food insecurity, safe drinking water, and cultural nutrition and physical activity patterns as well as environmental and policy contexts that influence the risk. Efforts may need to include more attention to upstream determinants of health or attributes of the communities where the populations with the highest burden live. The findings linking neighborhood features to one’s health status illustrate how a community can influence risk of many chronic health conditions, including obesity. For example, a study of neighborhoods in 3 US metropolitan regions (San Diego, Seattle, and Baltimore) from 2009 to 2010 assessed pedestrian environment features for walkability factors (eg, density). The study found that “across all three regions, low-income neighborhoods and neighborhoods with a high proportion of racial/ethnic minorities had poorer aesthetics and social elements (eg, graffiti, broken windows, litter) than neighborhoods with higher median income or fewer racial/ethnic minorities” (20). Likewise, if marketing of unhealthy products and/or fast-food establishments are unequally distributed across a community or are clustered near schools, communities may consider addressing this issue paired with improving healthy offerings (16,23,24). For individuals from the groups with the largest disparities, it is also important to focus attention on enhancing access to and reimbursement for quality health care services for growth assessment and obesity screening, and for persons with obesity and disease risk, appropriate referral to evidence-based healthy weight or prediabetes management programs and other treatment modalities (25,26).

In isolation, DNPAO resources, equivalent to $0.31 investment per American per year, will not be able to prevent obesity among at-risk Americans nor reduce the racial and ethnic disparities in the national burden of obesity. In addition to public health, many partners are needed, including policy makers, state and local organizations, business and community leaders, ECE, schools, industry, federal agencies, health care systems and providers, payers, faith-based organizations, community planners, food growers and distributors, families, and individuals. Using combined approaches, these partners should strive to best improve the ability to prevent obesity and its consequences for those with the burden. Such multisector partnerships can create positive changes at the community level to promote healthy eating and active living in areas where individuals may be at risk for obesity because of where they live and work. These focus areas could include making it easier for families with children to buy healthy, affordable foods and beverages near their homes; helping to provide access to safe, free drinking water in places such as community parks, recreation areas, child care centers, and schools; helping local schools open up gyms, playgrounds, and sports fields during nonschool hours so more children can safely play; increasing the number of safe and accessible sidewalks and bike paths to schools, parks and everyday destinations; and helping schools and ECE providers use best practices for improving nutrition and increasing physical activity. Demonstrated success in these approaches would be reductions in the disparities in upstream indicators (ie, improved community and behavioral determinants of health) and reductions in the obesity burden that is evident in CDC’s childhood obesity data and the maps above.

DNPAO is committed to supporting efforts to reduce racial and ethnic disparities in obesity by continuing to share what is working through partners and grantees, to develop tools that aid community engagement and the implementation of evidenced-based interventions, and to track obesity and its risk factors. Each sector and organization has a role to play in being part of the solution. To reduce the current disparities that exist in the burden of obesity, all parts of society need to relentlessly and intentionally work to address the causes of these disparities to help give all a fair chance at health.
